# Editorial: International perspectives on older adult social isolation and loneliness

**DOI:** 10.3389/fpubh.2025.1590229

**Published:** 2025-04-08

**Authors:** Lenard W. Kaye, James Lubben, Mercedes Bern-Klug, Ted K. S. Ng, Roger O'Sullivan, Matthew Lee Smith

**Affiliations:** ^1^Center on Aging, School of Social Work, University of Maine, Orono, ME, United States; ^2^School of Social Work, Boston College, Chestnut Hill, MA, United States; ^3^Luuskin School of Public Affairs, University of California - Los Angeles, Los Angeles, CA, United States; ^4^Aging and Longevity Studies Program, School of Social Work, University of Iowa, Iowa City, IA, United States; ^5^Department of Internal Medicine, Rush Institute for Healthy Aging, Rush University Medical Center, Chicago, IL, United States; ^6^Ageing Research and Development Division, Institute of Public Health, Dublin, Ireland; ^7^Bamford Centre for Mental Health and Wellbeing, University of Ulster at Belfast, Belfast, United Kingdom; ^8^School of Public Health, Texas A&M University, College Station, TX, United States

**Keywords:** social connection, older adult, social isolation, loneliness, international perspectives

## 1 Motivation for this Research Topic

This Research Topic was conceived by the editors as a vehicle for critically addressing the unprecedented and urgent global public health challenge posed by the escalating levels of isolation, loneliness, and disconnection experienced by older adults worldwide, especially against the backdrop of the COVID-19 pandemic.

Given the pervasive nature of social isolation and loneliness, we were committed to welcoming cross- and interdisciplinary contributions, making space for considerations about the influence of physical, environmental, social, psychological, cultural, and economic forces on late-life relationships and connectedness, and lack thereof, as conceived by an international roster of researchers and practice scholars. The perspectives of theorists, educators, administrators, public health officials, clinicians, and program planners have been included to ensure arriving at a more nuanced appreciation of what has been proclaimed as one of the greatest public health challenges of our day.

The editors of this Research Topic recognized the importance of giving voice to multiple perspectives about a research area that was considered crucial in arriving at a greater and more balanced understanding of the conditions that put older adults at risk of becoming socially isolated and lonely. More specifically, these include (a) the extent to which social isolation and loneliness are considered personal, community, and societal threats, in line with the socio-ecological model; (b) the wide range of impacts that the COVID-19 pandemic has had on individuals at greatest risk, its negative consequences on virtually all aspects of daily life; and (c) the steps that can be taken to prevent, reduce, and reverse its occurrence. Ultimately, this Research Topic intends to help us achieve a more thorough understanding of the underlying causes and correlates of older adult social isolation and loneliness as well as promising programmatic strategies for bolstering older adult social and emotional health and community engagement across diverse cultures, social systems, and populations.

We believe this Research Topic represents some of the most current theoretical, programmatic, and clinical research and evaluative assessments from around the world inquiring into the growing fragility of late-life relationships and the accompanying feelings of human disengagement. From multiple disciplinary and professional perspectives, this Research Topic serves to document our current understanding of the complexities surrounding the negative impacts of weakened relational ties on older adult safety, health, and wellbeing. It also demonstrates the application value of a range of research and evaluation methodologies, measurement strategies, and analytic approaches that can be employed when collecting both quantitative and qualitative data and scrutinizing them. At the same time, it showcases some of the most promising programmatic strategies and interventive techniques that show the greatest promise in helping to repair and maintain the integrity of an older adult's social and community network and support system.

## 2 Reflections about the Research Topic

The 54 papers in this Research Topic employ a broad range of methodologies including employing a variety of measures and sampling techniques. The Research Topic contains 40 original research papers, five reviews, three community case studies, a randomized clinical trial, a methods paper, a conceptual framework paper, a perspective paper, and an opinion piece. Of the original research papers, the majority analyzed quantitative data, two used qualitative data and three used mixed methods. Papers reporting results from cross-sectional data outnumber longitudinal papers. In fact, there are twice as many cross-sectional research papers compared to papers analyzing longitudinal data.

Papers reporting quantitative results from secondary analyses outnumber primary analysis empirical papers three to one. Four secondary datasets from China (CLASS, CLHLS, CHARLS, and ICFPS) were tapped for papers as were five datasets from the United States (HRS, HAPID, NSHAP, Rush MAP, and NSOAAP). Secondary analytic techniques were also applied to datasets from the Republic of Korea, Germany, Northern Ireland, Norway, and Sweden. Respondents from 18 European countries are featured in the SHARE dataset.

This Research Topic of papers includes authors with institutional affiliations from 14 countries: Australia, Canada, China, Germany, Ireland, Japan, New Zealand, Norway, Republic of Korea, Romania, Sweden, Switzerland, United Kingdom, United States. Respondents from 29 countries (including the 18 in the SHARE dataset) are represented among the papers.

Unfortunately, many countries are not represented in this Research Topic, including India, Russia, the countries of Africa, as well as those in Central and South America. Without research emanating from these regions of the world, our understanding of loneliness and social isolation will remain incomplete.

Slightly less than half of the articles in this Research Topic report data that were collected pre-COVID pandemic, and 18 report data collected during the pandemic. Another three articles conducted data collection both prior to and during the COVID pandemic. Three articles reflect data collected post-pandemic.

Most of the data in this Research Topic were collected from older adults living in the community, with only a few specifically mentioning the inclusion of individuals living in nursing homes and other long-term care communities. Most of the studies did not indicate whether people with cognitive impairment were included. Of the 16 that did mention they considered cognitive impairment, about half indicated they included persons with cognitive impairment in the sample, but not those living with severe or advanced Alzheimer's disease or related dementias.

A variety of social isolation and loneliness measures were utilized in this study collection. In terms of measuring loneliness, some studies asked how frequently the respondent felt lonely in the past week or used a standardized tool such as the UCLA Loneliness Scale (1) or the De Jong Gierveld Loneliness Scale (2). Social isolation was also measured in a variety of ways including asking individuals whether they had a confidante, asking about their level of social support, having them complete an ego-centered, social network map, asking how frequently they participated in various social activities, or through administration of a standardized measurement tool such as the Lubben Social Network Scale (3–5). In many studies, respondents were asked about their living arrangements, and the data were converted into a dichotomous variable (living alone - yes or no). The article by Smith and Barrett in this Research Topic proposes use of a more recently developed measurement tool, the Upstream Social Interaction Risk Scale (U-SIRS-13), which was also incorporated in the community case study of a multi-sector collaboration by Marcos et al..

In approximately half of the articles, a named theory was explicitly mentioned as undergirding the study and inspiring the inclusion of a question or series of questions in the data collection protocols that were developed. It would benefit this field of study if the use of theory was consistently employed to inform the planning and undertaking of future research endeavors. In other words, it is encouraged that use of theory be explicitly brought to bear in terms of informing study design, guiding data collection and analysis, and then incorporated into the meaning making of findings during discussion.

## 3 The current state of social isolation and loneliness scholarship

Scholarship represented in this Research Topic indicates that differences remain in the extent of history, theory, conceptual grounding, and overall development of social isolation vs. loneliness scholarship with the literature on social isolation still situated at an earlier stage of evolution.

We find that there is continued conflation of these related yet distinct constructs. It is critical that we come to a more precise understanding of the overlaps and distinctions between social isolation and loneliness. Though recent studies have increasingly recognized their differences, we still come across studies that commingle these two constructs. This is becoming an even more relevant issue in today's world as the COVID-19 pandemic has shone a spotlight on the importance of these two constructs. As a result, there has been an influx of researchers and practitioners who are focusing their attention on social isolation and loneliness. There are also other constructs related to social connectedness that are also seemingly conflated with social isolation and loneliness, including social engagement, social network, social activities/involvement, and social support. Since this Research Topic focused on social isolation and loneliness, we will not elaborate further on these other related constructs.

There remains a lack of consensus about preferred measures for social isolation and loneliness. By definition, social isolation is defined as having few social relationships or infrequent social contact with others, and loneliness is defined as a negative feeling resulting from the subjective experience of perceived unfulfilled social, emotional and intimate needs, feeling left out, and the lack of a sense of belonging at a local or societal level (6). A recent WHO report offers the following distinction between the term's social isolation and loneliness: “Social isolation and loneliness are forms of social disconnection. The former is the objective state of having few roles, relationships or social interactions, and constitutes the structural dimension of social disconnection. The latter is more subjective, i.e., the unpleasant or negative feeling/emotion resulting from perceived lack of social connection, reflecting a discrepancy between desired and actual experience of connection” (7).

To further advance knowledge and understanding about social isolation and loneliness, the extent to which consensus needs to be reached on measures for social isolation and loneliness across research fields, countries, cohorts, and stakeholders should be further determined. The advantages and disadvantages of achieving broad scale measurement tool agreement need to be considered, including the extent to which consensus across research studies impacts the soundness of the underlying constructs claiming to be measured, hinders comparisons of findings across cohorts and countries, etc. Multiple editors of this Research Topic have examined and provided recommendations related to this Research Topic. For example, a recent opinion piece reviewed the status of measures of social isolation among older populations and provided guidance to the research community (8). Fried et al. (9) previously called for a unified approach to the study of loneliness and a greater consensus on the definitions and measures of loneliness to help support those designing and delivering policy and services. More recently, an inventory of existing social connection measures was compiled to provide the research community with validated measurement options for research and practice (10).

There is a scarcity of studies examining trends and comparisons within and across countries, as well as across time periods and generational cohorts. Within countries, sub-population level nuances need to be better understood including those subgroups/subpopulations at greatest risk, as well as possibly underserved communities such as rural older adults, racial and ethnic minorities, etc. Across countries, more comparisons are also needed to better understand variations in population-level prevalence, incidence, and related macro-level differences. Across time periods and/or generations, research is needed to assess cohort effects, including longitudinal follow-ups of the same individuals over an extended period (ideally from mid-life or earlier to late-life).

Social isolation and loneliness are associated with the pertinent outcomes of interest and many other variables that could confound the associations examined. Hence, a sufficient control for confounders in research studies is essential. This point is illustrated by Victor (11). She highlighted that there is a need to minimize residual confounding effects, as many studies examining the associations between loneliness and cognition did not control for measures of social connection and isolation, depressive symptoms, and other pertinent confounders.

Continued research about the impact of the COVID-19 pandemic and its compounding detrimental effects on the physical and mental health of older adults [especially cognition, cognitive impairment, and Alzheimer's Disease and Related Dementias (ADRD)] as it relates to the experience of social isolation and loneliness warrants systematic study. Greater precision is needed in terms of distinguishing between cause and effect influences as opposed to associations among key health variables and the conditions of social isolation and loneliness (12). The impacts of COVID-19 and how the pandemic has altered how we interact with each other over the long-term are questions yet to be fully understood (13). For example, remote and hybrid work arrangements and switching to interacting with friends and families online have become way more common. COVID-19 has also been linked to an increased risk of Alzheimer's Disease and Related Dementias (14, 15). Unsurprisingly, social isolation and loneliness are two prominent risk factors preceding the development of ADRD (16–19). Alarmingly, the combined effects of COVID-19 and social isolation and loneliness can be even more pronounced than either of them alone, especially on ADRD. Though emerging studies have shown evidence substantiating this link (15, 20), cognitive outcomes require an extended follow-up period, especially in cognitively healthy older adults. Longitudinal follow-up studies spanning decades, which incorporate measures of social isolation and loneliness, and measures related to COVID-19, are needed to understand the long-term intertwining effects of COVID-19 and social isolation and loneliness on older adults' health, particularly cognitive outcomes (Lawlor et al.).

Finally, a scarcity of effective programmatic interventions to ameliorate social isolation and loneliness that have been systematically tested. This requires the contributions of interdisciplinary and cross-disciplinary professions and disciplines including sociology, social work, psychology, medicine (especially psychiatry and neurology), as well as deep knowledge of randomized controlled trials (RCTs), public health research methodologies, and more. Such research inquiry must not reflect a siloed mentality. Cross-fertilization of ideas and the use of big data that incorporates measures central to different fields yet related to social isolation and loneliness are strongly encouraged as are study designs that involve the community (i.e., community-based participatory research).

## 4 Recommended research directions

The editors of this Research Topic reached a consensus about the importance of the following future research directions that will further our understanding of the risk and protective factors, detrimental effects, particularly impacts on mortality (21, 22) and cognitive impairment (16, 23), as well as potential interventions that could ameliorate the negative consequences of older adult social isolation and loneliness.

Recommended avenues for future social isolation and loneliness research include efforts that:

- Focus on solutions at both the individual or clinical level and at the community and societal level, across various levels as indicated in the socio-ecological model (24). For example, an article by Smith et al. (25) offers nine actionable community- and societal-level strategies to strengthen community capacity and promote cross-sectoral support for social connection among older adults (e.g., establish common nomenclature, use common measures, strengthen referral pathways, expand evidence for programs and services, leverage funding).
- Test the value and efficacy of differing measurement tools and definitions of the social isolation and loneliness constructs. Clearly, we have yet to arrive at a consensus on these matters and need to consider how to send a more coherent message to those designing and conducting research studies as well as developing and implementing policy and services. For example, an international meeting on loneliness was held in Belfast, Ireland in December 2018 that developed a consensus statement regarding key issues for moving forward research and clinical practice on loneliness (26). Pomeroy and associates (8) suggested convening an international meeting on social isolation like the Belfast 2018 meeting on loneliness. It would seem timely to consider an international meeting to build consensus on measurement and coherent messaging for social isolation and loneliness, involving various stakeholders, including policy makers and academics from diverse disciplines. This effort is particularly pertinent given the surge in interest from the research, service, and clinical communities concerning the detrimental effects of social isolation and loneliness on a plethora of health outcomes.
- Address the debate on whether loneliness levels are increasing or not. Compelling data are needed—not just at a population level and across different countries that have distinct population demographics, but also at a subpopulation level to allow intra-country comparisons.
- Recognize social isolation and loneliness are public health issues—what can we learn from other public health interventions? The recent World Health Organization statement (7) regarding mental health and social connection is relevant here. Furthermore, the 2023 Report from the U.S. Surgeon General's report on Our Epidemic of Loneliness and Isolation is pertinent in this regard as well (27).
- Investigate the question: “are we destined to increasingly be a society of loners?” This is a relevant question to ask and pursue, especially against the backdrop of population aging and reduced fertility rates across countries that have resulted in the inverted pyramid population age composition/structure.
- Apart from inflammatory markers (28, 29), are there other emerging biological correlates/signatures of social isolation and loneliness? There has been preliminary evidence of inflammatory markers (30–32), DNA methylation clock (33), and depressive symptoms (34) being the mediators linking social isolation and loneliness to cognitive decline/impairment. Are there other potential mediators that are also modifiable via interventions?
- More precisely analyze the relationship between social isolation and loneliness and cognitive decline/impairment and ADRD (11), including the extent to which these constructs are causative, symptomatic, comorbidities, or something different.
- Better explain why interventions targeting social isolation and loneliness seem, all too often, to fall short of their intent. Too few studies offer rigorous models of implementation and evaluation in this regard. There are exceptions, including recent trials led by Dodge et al. (35), that have shown preliminary evidence of an intervention, the Internet-Based Conversational Engagement, for older adults facing social isolation, improving cognition and metrics of mental health. Additionally, Ng et al. (36, 37) have shown, in a preliminary RCT, that horticultural therapy with older adults who are cognitively healthy improved their degree of social connectedness, with its effect mediated by a prominent inflammatory marker, the interleukin-6. Lastly, Creswell et al. (38) conducted an RCT on mindfulness-based stress reduction training, which reduces loneliness and pro-inflammatory gene expression in older adults. Replication/validation studies of these and other interventions in different populations are needed.
- Consider whether it is time for a coordinated global strategy on addressing social isolation and loneliness. How do we ensure contributions and perspectives from underrepresented countries and populations are considered? Further, how are the findings gleaned across culturally diverse regions generalizable to or possibly different within populations? Are findings and interventions “one-size-fits-all” or are nuances observed and hence interventions need to be tailored to different populations? Efforts to harmonize and synergize global efforts are underway (39, 40); however, they would benefit from additional governmental and cross-sectoral support fuelled by rigorous surveillance and evidence about the effectiveness of social connection programs and services.

## 5 Conclusion

Perhaps not surprisingly, the impressive compendium of contemporary research and scholarship on older adult social isolation and loneliness included in this Frontiers Research Topic has raised as many questions as it has answered. As a result, we have shared our thoughts on what remains as part of the unfinished research agenda when it comes to better understanding and responding to what we perceive to be these two major contemporary threats to individual, community, and societal health, and wellbeing across the globe.

The number, scope, breath, and quality of the contributions that we received speak volumes to the timeliness and significance of the topic in the public health community and the growing level of concern and interest surrounding the interpretation of both constructs. We sincerely hope that this Research Topic further sparks investigative efforts, broadly conceived, at comprehending and responding more fully to the deeply concerning impacts of social isolation and loneliness on older adults and the world in which they live.
